# RanGAP1 accumulates in stress-induced cytoplasmic compartments that are distinct from stress granules and P-bodies

**DOI:** 10.17912/micropub.biology.002003

**Published:** 2026-03-16

**Authors:** Justin Mezzanotte, Jessica Shi, Ursula Stochaj

**Affiliations:** 1 Physiology, McGill University, Montreal, QC, CA; 2 Quantitative Life Sciences Program, McGill University, Montreal, QC, CA

## Abstract

Stress granules (SGs) and P-bodies are dynamic compartments in the cytoplasm of eukaryotic cells. SGs assemble in response to stress. The nuclear transport factor RanGAP1 is linked to the pathologies of several human diseases. RanGAP1 concentrates at nuclear pore complexes, but can also associate with cytoplasmic biomolecular condensates. The small molecule pifithrin-µ (PFT-µ) inhibits Hsp70 family members and has potential as a therapeutic agent. PFT-µ triggers SG formation in human cancer cells. This prompted us to evaluate the subcellular localization of RanGAP1 upon exposure to PFT-µ. We show that RanGAP1 accumulates in unique cytoplasmic compartments that do not colocalize with SGs or P-bodies.

**Figure 1. Impact of PFT-µ treatment on RanGAP1 subcellular localization and SUMOylation:**
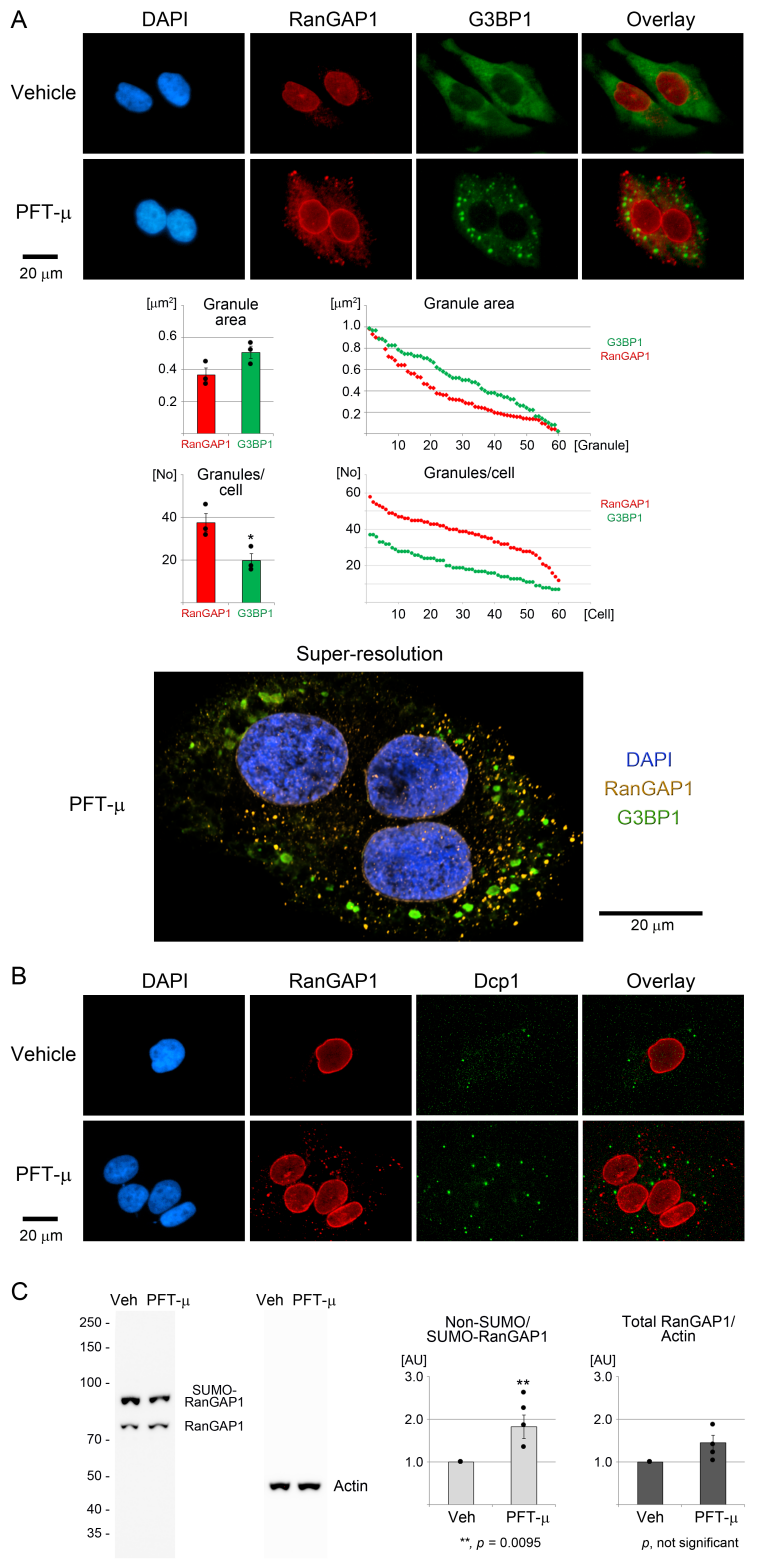
(A-C) Cells were treated with the vehicle DMSO or PFT-µ for 2 h. Samples were then processed for immunostaining or Western blotting. (A-B) Images were acquired by conventional fluorescence or super-resolution microscopy, as indicated. Size bars are 20 µm. (A) RanGAP1 was detected by immunostaining; G3BP1 was used as SG marker. Granule parameters were quantified for three independent experiments. The granule area in µm ^2^ was measured for 20 granules per experiment. The number of granules per cell [No] was determined for 20 cells for each experiment. Bar graphs depict the averages ± standard error of the mean. Student’s t-test revealed significant differences for the number of granules/cell; *, *p* < 0.05. Scatter plots display results for individual granules or cells. (B) RanGAP1 biomolecular condensates were compared with P-bodies, using the marker protein Dcp1. (C) Western blotting of crude cell extracts evaluated the SUMOylation and abundance of RanGAP1. A representative blot of four independent experiments is shown. The molecular mass of marker proteins is depicted at the left margin. The ratio non-SUMOylated/SUMOylated RanGAP1 was normalized to vehicle controls (light gray bars). The abundance of total RanGAP1 (SUMOylated RanGAP1 + non-SUMOylated RanGAP1) relative to actin is displayed as dark gray bars. Data are shown for four independent experiments as averages ± standard error of the mean. Significant differences were identified with Student’s t-test; *, *p* < 0.05; **, *p* < 0.01. Veh, vehicle; PFT-µ, pifithrin-µ; AU, arbitrary units.

## Description

Stress granules (SGs) are dynamic, membrane-less biomolecular condensates that are assembled when cells experience deleterious growth conditions. In eukaryotes, SGs serve important functions in the regulation of mRNA metabolism and the subcellular localization of proteins (Mahboubi & Stochaj, 2017). SGs are heterogeneous in composition and their properties are in part determined by the stressor (Advani & Ivanov, 2020). SGs are often located adjacent to P-bodies, a different type of biomolecular condensate that regulates the fate of translationally repressed mRNAs. Unlike SGs, P-bodies are present under non-stress and stress conditions (Chin Sang et al., 2025). Identifying the proteins that localize to SGs and P-bodies is critical for defining granule functions and their contributions to cellular stress responses, especially in the context of neurodegeneration and cancer (McGoldrick et al., 2023; Zhou et al., 2023).

RanGAP1 is a key regulator of nucleocytoplasmic transport (Takeda et al., 2005). The protein is required for classical nuclear import and the dismantling of Crm1-dependent nuclear export complexes in the cytoplasm (Hutten et al., 2008; Ritterhoff et al., 2016). Interphase cells contain two pools of RanGAP1, RanGAP1 located in the cytoplasm and SUMOylated RanGAP1 associated with the nuclear pore complex (Mahajan et al., 1998; Matunis et al., 1998). Notably, the presence of RanGAP1 at the nuclear envelope may not be required to support nuclear transport in cultured cells (Chen et al., 2021). This suggests a complex relationship between RanGAP1 localization and function. Our previous work showed that oxidative stress does not significantly change the association of RanGAP1 with the nuclear envelope (Crampton et al., 2009).

The subcellular distribution of RanGAP1 is relevant to human health, and links have been established to various diseases. In particular, RanGAP1 interacts with the peptides generated by translation of C9orf72 repeats, which leads to the cytoplasmic mislocalization of RanGAP1 (Jafarinia et al., 2024; Ryan et al., 2022; Zhang et al., 2015). RanGAP1 is also mislocalized in other models of neurodegeneration (Plessis-Belair et al., 2024).

A loss of RanGAP1 protein occurs in gastric cancer induced by *H. pylori* infection and lymph node metastases derived from these tumors (Zhou et al., 2016). As well, RanGAP1 can decline in abundance in human osteosarcoma cells (Gong et al., 2023).

Different nuclear transport factors accumulate in SGs (Mahboubi & Stochaj, 2017). For instance, members of the importin-α and importin-β families, regulators of the RanGTPase, and nucleoporins associate with SGs that are generated upon oxidative stress. Notably, RanGAP1 is absent from arsenite-induced SGs (Zhang et al., 2018), but present in other cytoplasmic condensates (McGoldrick et al., 2023).

Pifithrin-µ (PFT-µ, also known as PES) is a small molecule that covalently modifies members of the hsp70 family, which alters their interactions with several co-chaperones (Leu et al., 2009; Yang et al., 2021). This is exemplified by diminished binding of Hsp40, CHIP, and Bag-1M. The importance of molecular chaperones for overall proteostasis, biomolecular condensate formation, neurodegeneration, and all aspects of cancer cell biology is well established (Kohler & Andréasson, 2023; Lang et al., 2019; Singh et al., 2025). These links make PFT-µ and its derivatives promising compounds for the development of new anti-cancer treatments.

Our previous work showed that PFT-µ induces the formation of conventional SGs (Mahboubi et al., 2024). Here, we expanded the knowledge pertinent to the effects of PFT-µ on cytoplasmic compartmentalization. In particular, we investigated the impact of PFT-µ on the subcellular localization of RanGAP1. The events were studied in HeLa cells, because they are a commonly used model system to assess the formation of SGs (Mahboubi et al., 2024) and other biomolecular condensates.

Figure 1A evaluates the RanGAP1 distribution in HeLa cells after treatment with vehicle (DMSO) or PFT-µ. The granule-nucleating protein G3BP1 was used as SG marker, and Dcp1 demarcated P-bodies. PFT-µ robustly induced the formation of SGs after 2 h. Interestingly, PFT-µ led to the accumulation of RanGAP1 in cytoplasmic compartments that were irregularly shaped and devoid of G3BP1. The failure of RanGAP1 to concentrate in PFT-µ SGs is consistent with the observations for arsenite-induced SGs (Zhang et al., 2018). The quantification of granule size and the number of granules/cell revealed that on average RanGAP1 granules were smaller in size than PFT-µ-induced SGs ( Fig. 1A ). In addition, RanGAP1 containing biomolecular condensates were significantly more numerous than SGs. Nevertheless, our analyses at the single granule or single cell levels revealed that both granule size and the number of granules/cell varied widely ( Fig. 1A ).

To obtain a higher resolution view of the RanGAP1 distribution in the cytoplasm, we conducted super-resolution microscopy on PFT-µ-treated cells ( Figure 1A ; animation in the Supplemental File). Super-resolution images verified that RanGAP1 and G3BP1 reside in separate locations. The cytoplasmic compartments accumulating RanGAP1 were smaller than SGs. Accordingly, it was possible that RanGAP1 located to P-bodies. However, immunolocalization of P-bodies with the marker protein Dcp1 demonstrated that RanGAP1 did not concentrate in P-bodies ( Figure 1B ).

RanGAP1 occurs as SUMOylated and non-SUMOylated forms, which differ in electrophoretic mobility in denaturing gels. Figure 1C assessed RanGAP1 in control and PFT-µ-treated samples by Western blot analysis of crude cell extracts. Quantitative analysis revealed that the ratio of non-SUMO-RanGAP1/SUMO-RanGAP1 significantly increased upon PFT-µ incubation. Although not formally tested by us, this increase may suggest that the association of RanGAP1 with cytoplasmic granules is linked to the removal of the SUMO moiety from RanGAP1. Interestingly, the abundance of total RanGAP1 (SUMOylated + non-SUMOylated) was not significantly changed by PFT-µ treatment ( Figure 1C ).

Studies by others emphasize the diversity of cytoplasmic biomolecular condensates that concentrate nuclear transport factors. For example, granules containing importin-β1 are present in neurons of *C9orf72* knockout mice that display pathologies of neurodegeneration (McGoldrick et al., 2023). While RanGAP1 associates with importin-β1 granules, these compartments also colocalized with G3BP1. Therefore, the RanGAP1 granules induced by PFT-µ are unique; they differ in composition from previously described biomolecular condensates.

Taken together, our results demonstrate that the heterogeneity of granules that accumulate nuclear transport factors goes beyond the different forms of SGs and P-bodies. These data highlight the need for future studies that define the biological relevance of the diverse types of cytoplasmic biomolecular condensates that are present under various physiological conditions.

## Methods

**Cell culture and treatment**

HeLa cells were cultured and incubated with the vehicle DMSO or 50 µM PFT-µ for 2 h, essentially as described (Mahboubi et al., 2024). The final concentration of the vehicle DMSO was 0.1% (vol/vol) for all conditions.

**Immunofluorescence staining, fluorescence microscopy**

Following the incubation with vehicle or PFT-µ, cells were fixed, processed for immunostaining, and imaged by conventional fluorescence microscopy as published by us (Mahboubi et al., 2024).

**Measurement of granule area and the number of granules per cell**

Area measurements were conducted with the ImageJ Particle Analysis Tool (ImageJ, 2026). Manual counting determined the number of granules per cell.

**Super-resolution microscopy and image processing**

Cells were imaged with a Nikon AX R confocal super-resolution system with NSPARC (Nikon Instruments Inc., Tokyo, Japan) mounted on a Ti2 inverted microscope. Images were acquired using a 40× oil-immersion objective (NA 1.42). For z-stacks, 23 planes were acquired with a 0.2 µm step size. Excitation/emission settings were appropriate for DAPI (405 nm/429–474 nm), Alexa488 (488 nm/499–537 nm), and Cy3 (561 nm/585–625 nm). Emission was collected sequentially for different channels.

Acquisition parameters included resonant bidirectional scanning, 2× line averaging, zoom factor of 1.39, with a resolution of 2048 × 2048 pixels. The pinhole size was set to 32.6 µm for all channels.

**Preparation of crude cell extracts and Western blot analysis**

The generation of crude cell extracts and Western blot analysis followed our published procedures (Mahboubi et al., 2024).

## Reagents

**Materials**

Pifithrin-µ (A370437) was purchased from AmBeed (Buffalo Grove, IL); the compound was dissolved in DMSO.

**&nbsp;**

**Antibodies and dilutions**

**Table:** 

**Primary Antibodies**				
**Protein**	**Supplier**	**Catalog Number**	**Dilution for** **Western Blotting**	**Dilution for** **Immuno­localization**
Actin	Chemicon, Temecula, CA, USA	mab1501	1:100 000	NA
Dcp1	Santa Cruz Biotechnology	sc-100706	NA	1:200
G3BP1	Santa Cruz Biotechnology	sc-365338	NA	1:1 600
RanGAP1	Santa Cruz Biotechnology	sc-25630	1:1 000	1:400
**Secondary Antibodies**				
**Tag**		**Supplier**	**Dilution for** **Western Blotting**	**Dilution for** **Immuno­localization**
Horseradish peroxidase (HRP)		Jackson ImmunoResearch, West Grove, PA, USA	1:2 000	NA
Alexa Fluor ^®^ 488, Cy3		Jackson ImmunoResearch	NA	1:200

## Data Availability

Description: Animation of 3D reconstruction for super-resolution microscopy images.. Resource Type: Audiovisual. DOI: https://doi.org/10.22002/64wtx-86e28
